# Editorial: Interaction Between Modern Radiotherapy and Novel Drugs in Prostate Cancer: Future Perspectives

**DOI:** 10.3389/fonc.2022.876318

**Published:** 2022-03-30

**Authors:** Beatrice Detti, Thomas Zilli, Gianluca Ingrosso, Ivone Ribeiro, Andrea Lancia

**Affiliations:** ^1^ Azienda Ospedaliera Universitaria Careggi, Radiotherapy Unit, Firenze, Italy; ^2^ Radiation Oncology, Geneva University Hospital, Geneva, Switzerland; ^3^ Radiation Oncology Section, Department of Medicine and Surgery, University of Perugia, Perugia, Italy; ^4^ Radiation Oncology, University Hospital of Gran Canaria Dr. Negrin Las Palmas de Gran Canaria, Las Palmas, Spain; ^5^ Radiation Oncology, San Matteo Hospital Foundation (IRCCS) Pavia, Pavia, Italy

**Keywords:** Stereototactic Body Radiation Therapy (SBRT), poly-ADP-ribose polymerase inhibitors (PARP-I), androgen receptors inhibitors, prostate-specific membrane antigen (PSMA) positron emission tomography, metastasis-directed therapies (MDT)

Advanced prostate cancer currently represents one of the most exemplary scenarios of a true multimodal approach, in which the efforts of traditional local therapies such as surgery and definitive radiotherapy (RT) must be necessarily reinforced by the adoption of systemic therapies, mostly hormonal treatment strategies.

Despite this holistic attitude towards the disease has historically found its sweet spot for high-risk localised or locally advanced hormone-sensitive prostate cancer, we are witnessing the emergence of an era of combined tailored approaches also in subsequent evolutionary disease phases, namely in the castration resistant prostate cancer (CRPC) setting. This broader vision has been made possible thanks to several factors, starting from a deeper knowledge on the underlying processes of acquired resistance to androgen deprivation therapy (ADT), disease progression, and eventually metastatization. In addition to these biological insights leading to the development of new molecules progressively expanding the drug armamentarium at our disposal, tremendous strides have been made also in the field of radiotherapy, with the increasing implementation of Steretotactic Body Radiation Therapy (SBRT) and the rapidly expanding field of particle therapies. The success of such techniques goes hand in hand with the improvements in terms of imaging modalities for disease staging and restaging; in this setting, the advent of prostate-specific membrane antigen (PSMA) positron emission tomography (PET) may represent a momentous game changer not only in terms of early diagnosis but also for disease management, as suggested by the experience of Fan et al. in this Research Topic.

Marvaso et al. provide us some insights concerning the state of the art in the treatment of high-risk prostate cancer: currently the different guidelines agree on the role of a radical management approach, namely with radical prostatectomy or definitive external beam radiotherapy (EBRT) combined with long-course ADT. Among the new scenarios for a future multimodal tailored approach, we soon will be able to widely support with mature data hypofractionated and ultra-hypofractionated RT, new drugs (Androgen Receptors Inhibitors, ARIs and Poly-ADP-ribose polymerase inhibitors -PARP-I), and particle therapy, increasing the therapeutic options and improving the oncological outcomes of this category of patients.

ARIs are already a new important treatment option in the management of non-metastatic CRPC disease. Based on the three published randomized clinical trials SPARTAN, ARAMIS and PROSPER (1–3), addition of ARIs to standard ADT is associated with significant improvements in metastasis-free survival and overall survival reduction compared to standard of care treatments.

Poly-ADP-ribose polymerase inhibitors (PARP-I) are widely used in several tumor lines, and are shown to have a chemo-sensitizing effect on tumours that have developed defects and mutations in proteins involved in the control and repair of DNA damage, such as p53, ATM, MRE11 and BRCA1-2 (4–6). The use of these new molecules is currently the subject of numerous studies, but their efficacy and safety in combination with standard RT treatment has not yet been clearly demonstrated.

Otherwise, when it comes to patients with an oligometastatic disease (< 3/5 lesions), metastasis-directed therapies (MDT) may represent an appealing opportunity for treating patients in this setting as demonstrated by retrospective series and two randomized prospective trials (7–13).

Massaro et al. conducted a retrospective analysis investigating whether a local ablative radiotherapy (SBRT and 3DCRT) may represent a valuable strategy to maintain oligoprogressing patients in a mCRPC phase under ARI treatment. Overall (OS) and progression free survival (PFS) rates were significantly better if MDT was performed after 6 months rather than within 6 months from beginning of ARIs (Median OS: 45.5mo if RT > 6mo vs 23.4mo if RT > 6mo, p = 0.009; Median PFS: 30mo if RT > 6mo vs 9.2mo if RT > 6mo, p = 0.006).

Also in this scenario, to further delay metastatic progression and interfere with the natural course of the disease, integrating ARIs with SBRT targeting loco-regional lymph nodes and/or oligoprogressive disease could represent a valid choice of treatment. It seems to emerge from data also that adding SBRT to an ongoing systemic treatment may keep the disease controlled by eradicating the few cellular subclones that developed resistance during this oligoprogressive phase (14, 15).

Another option available for dealing with prostate cancer is particle therapy, most commonly carbon ion radiotherapy and proton beam therapy. These are promising alternative for treating prostate cancer, and the available evidences demonstrated a lower incidence of urinary and gastrointestinal toxicity compared with standard photon radiotherapy. However, the current quantity and quality of the available data are insufficient and controversial and the integration with systemic therapies are unclear (16).

Petersen and Hoyer in their literature review of 15 studies, with a grand total of 3’519 patients receiving ADT combined with particle therapy, pointed out that did not seem to be concrete evidence in literature about efficacy-related differences between adding ADT to particle therapy comparing to adding it to standard photon-based treatments.

Also from this review, it appears clear that ADT not only represents the therapeutic backbone in the context of metastatic disease, but it has also a key role in unfavourable intermediate- and high-risk localised prostate cancer patients. As a matter of fact, the majority of prostate cancer patients receive hormonal manipulation throughout their disease course. However, we as clinicians must be well aware of the side effects profile connected with this therapy, including the least known but potentially relevant consequences in terms of impaired quality of life.

Achard et al. analysed in their review the available data about the potential increased risk of dementia/Alzheimer’s disease in prostate cancer patients treated with long-term hormonal manipulation. Noteworthy, evidence suggests a correlation between ADT and both amyloid beta accumulation and hyperphosphorylation of the Tau protein in experimental models, although the molecular mechanism is yet unknown. However, studies reported so far on this Research Topic are retrospective and observational and require further prospective validation due to the large variability in the type of biomarkers analysed (and no amyloid or tau listed among them), ADT treatment duration, and cognitive outcomes.

Another promising breakthrough for CRPC has been made from a diagnostic point of view with PSMA-PET, which has been increasingly used in the monitoring and diagnosis of patients in a CRPC stage. In fact, a large number of studies investigated the possibly determinant role of PSMA-PET, which keeps demonstrating a higher sensitivity and specificity, especially in nmCRPC patients compared with conventional imaging (17–21); PSMA-PET could moreover induce a stage migration -mainly towards an oligometastatic state- in patients otherwise wrongly considered as M0.

We are facing 2022 with the awareness that combined approaches in the setting of advanced prostate cancer are here to stay. Treatments combining ADT and RT with new molecules such as ARIs undoubtedly represent the new future not only in high-risk localized prostate cancer but increasingly in the more advanced stages of disease. However, the optimal timing, the duration and the best therapeutic combination are still points to be investigated (22–25).

## Author Contributions

BD: conceptualization, writing, validation. GI: conceptualization, validation. AL: conceptualization, writing, validation. TZ: conceptualization, writing, validation. IR: validation. All authors listed have made a substantial, direct and intellectual contribution to the work, and approved it for publication.

## Conflict of Interest

The authors declare that the research was conducted in the absence of any commercial or financial relationships that could be construed as a potential conflict of interest.

## Publisher’s Note

All claims expressed in this article are solely those of the authors and do not necessarily represent those of their affiliated organizations, or those of the publisher, the editors and the reviewers. Any product that may be evaluated in this article, or claim that may be made by its manufacturer, is not guaranteed or endorsed by the publisher.
